# Emerging pathogenetic mechanisms in adolescent idiopathic scoliosis: the role of inflammation and gut microbiota

**DOI:** 10.1186/s13018-026-06837-w

**Published:** 2026-04-01

**Authors:** Bartosz Kruk, Karolina Skonieczna-Żydecka, Wiktoria Czarnecka, Natalia Tuczyńska, Albert Podkówka, Piotr Szredzki, Grzegorz Pasternak, Karolina Kokot, Jowita Biernawska, Sławomir Zacha

**Affiliations:** 1 Department of Pediatric Orthopedics and Traumatology, Pomeranian Medical University in Szczecin, Unii Lubelskiej 1, 70-252 Szczecin, Poland; 2 Department of Biochemical Science, Pomeranian Medical University in Szczecin, Broniewskiego 24, 71-460 Szczecin, Poland; 3 Collegium Medicum, University of Rzeszow, Rejtana 16C, 35-959 Rzeszów, Poland; 4 Department of Anaesthesiology and Intensive Care, Pomeranian Medical University, Unii Lubelskiej 1, 71-252 Szczecin, Poland

## Abstract

**Purpose:**

This narrative review was conducted to analyse current evidence on inflammation-related mechanisms contributing to the pathogenesis and progression of adolescent idiopathic scoliosis (AIS).

**Methods:**

A comprehensive narrative synthesis of studies investigating inflammatory biomarkers, immune cell phenotypes, cytokine pathways, paraspinal muscle immunobiology, gut microbiota composition, and their mechanistic links to bone and muscle remodeling in AIS was performed. Evidence was integrated from clinical, genetic, histological, microbiological, and experimental models.

**Results:**

Evidence indicates that AIS is associated with chronic low-grade inflammation affecting systemic immunity, bone metabolism, and paraspinal muscle structure. Altered cytokine activity (IL-6, IL-1β, TNF-α, IL-17) promotes osteoclastogenesis, extracellular matrix degradation, and reduced bone mineral density. Paraspinal muscles on the concave side exhibit fibrosis, macrophage imbalance, and impaired regeneration, consistent with persistent inflammatory signalling. Additionally, gut microbiota dysbiosis—characterized by reduced bacteria producing short chain fatty acids (SCFA) and increased pro-inflammatory taxa—may contribute to endotoxemia, immune activation, and disruption of the gut–bone–muscle axis. Inflammatory markers such as the neutrophil-to-lymphocyte ratio correlate with curve severity, and genetic and Mendelian randomization analyses suggest that specific microbial taxa may modulate AIS risk.

**Conclusions:**

Current evidence supports a multifactorial biological model of AIS in which chronic low-grade inflammation acts as a central integrator of systemic and local pathogenic processes. Altered cytokine signaling and immune cell imbalance promote dysregulated bone remodeling via the RANKL/RANK pathway, while persistent inflammatory activation within paraspinal muscles contributes to fibrosis, impaired regeneration, and biomechanical asymmetry. In parallel, gut microbiota dysbiosis may further amplify inflammatory signalling through intestinal barrier dysfunction, reduced production of anti-inflammatory microbial metabolites, and activation of the gut–bone–muscle axis. Although causal relationships remain to be fully established, these interconnected mechanisms provide a coherent framework linking immune dysregulation, musculoskeletal remodeling, and curve progression in AIS, highlighting opportunities for biomarker discovery and the development of targeted preventive and adjunctive therapeutic strategies.

## Introduction

Adolescent idiopathic scoliosis (AIS) is a three-dimensional spinal deformity diagnosed when the coronal plane curvature exceeds 10°, measured using the Cobb angle on standing radiographs [1, 2]. However, the Cobb angle is subject to intra- and interobserver variability, which should be considered in clinical practice [3]. Furthermore, conventional radiographic assessment reduces a complex three-dimensional deformity to two-dimensional projections, which may limit the accuracy of curve characterization. This discrepancy highlights the inherent challenges in quantifying spinal deformity and interpreting its progression in clinical and research settings [4].

Importantly, AIS is not only a structural deformity but also involves alterations in spinal kinematics and movement patterns. Early biomechanical studies demonstrated that abnormal rotational and composite movements are integral components of scoliotic deformity, providing additional insight into its three-dimensional nature [5].

Disease progression in AIS exhibits interindividual variability, underscoring its heterogeneous nature [6]. AIS is estimated to affect approximately 1–3% of the paediatric population, making it the most common spinal deformity among adolescents. The condition typically manifests between the ages of 10 and 16, with more severe forms being more frequently observed in females [7, 8]. Despite the diversity of proposed pathogenetic mechanisms, a unifying biological framework capable of linking skeletal growth, muscle dysfunction, and metabolic abnormalities in AIS remains to be established. Among these, alterations in hormonal regulation—particularly involving melatonin—have received considerable attention due to their potential impact on circadian rhythms, skeletal growth, and bone metabolism. However, current evidence remains inconsistent and does not support a clear causal relationship [9].

In this context, inflammation-related pathways have gained increasing attention in recent years as potential integrators of these processes. Chronic low-grade inflammation is now recognized as a key regulator of bone remodelling, muscle regeneration, and extracellular matrix turnover, even in conditions not classically defined as inflammatory. Importantly, inflammatory mediators and immune cells influence osteoclast–osteoblast coupling, muscle fibrosis, and tissue responses to mechanical loading—processes that are central to spinal stability and growth during adolescence [10].

Furthermore, emerging clinical and experimental data suggest that AIS may be associated with subtle but persistent immune activation rather than overt systemic inflammation. Alterations in inflammatory biomarkers, cytokine signalling, immune cell phenotypes, and paraspinal muscle immunobiology have been reported; however, these findings remain fragmented and insufficiently integrated within existing etiological models. Currently, advances in microbiome research have revealed gut microbiota–driven modulation of immune and metabolic pathways relevant to bone and muscle homeostasis, providing a novel perspective on AIS pathogenesis. Overall, these observations highlight inflammation-related mechanisms biologically plausible and timely focus for understanding AIS development and progression.

## Methods

The preparation of this narrative scoping review [11] followed several stages [12]. In the first stage, a broad research question was formulated to explore the role of inflammation-related mechanisms and gut microbiota alterations in the pathogenesis and progression of AIS. Next, a comprehensive literature search was conducted in the PubMed database using combinations of the terms “adolescent idiopathic scoliosis” with “inflammation,” “cytokines,” “immune cells,” “immune dysregulation,” “bone remodelling,” “paraspinal muscle,” “gut microbiota,” and “dysbiosis.” Systematic reviews, narrative reviews, observational clinical studies, genetic and experimental studies, as well as selected mechanistic animal and in vitro studies were included. Additional relevant publications were identified through manual screening of reference lists. Studies published in English were included from database inception up to August 2025. Study selection and narrative data synthesis were performed by the first and corresponding authors, followed by thematic collation, integration, and reporting of results.

### Inflammation and the role of immune cells in the pathogenesis of AIS

To provide a clearer theoretical framework, inflammation-related mechanisms implicated in AIS can be organized according to the primary tissues affected—namely systemic immune signalling, bone, and paraspinal muscle—while acknowledging their close biological interconnection. This tissue-based approach facilitates integration of cytokine pathways, immune cell activity, and downstream structural effects, thereby clarifying how chronic low-grade inflammation may contribute to spinal deformity development and progression.

#### Systemic inflammatory signalling and immune activation in AIS

Inflammation is a fundamental biological response that regulates tissue repair and homeostasis. While acute inflammation is typically self-limiting, chronic low-grade inflammation can persist and subtly disrupt tissue structure and function. In musculoskeletal tissues, immune cells and inflammatory mediators tightly regulate bone turnover, muscle regeneration, and extracellular matrix remodelling; dysregulation of these processes has been implicated in a range of skeletal disorders [13–15].

In AIS, accumulating evidence suggests the presence of subtle immune dysregulation rather than overt systemic inflammation. Several studies have reported alterations in inflammatory biomarkers and cytokine profiles in AIS patients, although many observations derive from perioperative settings where surgical stress—particularly elevation of IL-6—may confound interpretation [9]. Nevertheless, the recurring identification of inflammation-related signals has raised interest in their potential contribution to disease biology [15]. Among inflammatory pathways, the IL-17 axis has emerged as a candidate mediator of sustained immune activation. Activation of IL-17 receptors, including the IL-17RC subunit, triggers downstream signalling cascades that promote the production of pro-inflammatory cytokines, chemokines, and matrix metalloproteinases (MMPs). Through these effects, IL-17 signalling may amplify low-grade inflammation and facilitate tissue remodelling within spinal supporting structures.

Clinical evidence supporting systemic immune involvement in AIS also comes from hematological indices of inflammation. The neutrophil-to-lymphocyte ratio (NLR), a readily measurable marker of chronic inflammatory status, has been shown to correlate positively with Cobb angle severity. Elevated NLR reflects sustained neutrophil activation coupled with relative lymphopenia. This pattern suggests a shift toward innate immune dominance. Neutrophils release pro-inflammatory cytokines and proteolytic enzymes capable of influencing tissue remodelling, potentially contributing to curve progression. The lack of consistent associations with other indices, such as the monocyte-to-lymphocyte ratio, suggests selective involvement of specific inflammatory pathways in AIS [16, 17].

#### Inflammation-driven bone remodelling in AIS

In the context of musculoskeletal diseases, pro-inflammatory cytokines—primarily interleukin-1β (IL-1β), interleukin-6 (IL-6), and tumor necrosis factor-alpha (TNF-α)—play a critical role. These cytokines can exacerbate osteoclastogenesis, promote extracellular matrix degradation, and modulate bone metabolism, potentially leading to bone mass loss and weakening of supportive structures [18–22]. Anti-inflammatory cytokines, including interleukin 10 (IL-10) and transforming growth factor β (TGF-β), attenuate the inflammatory response by inhibiting the expression of genes encoding pro-inflammatory mediators and promoting the differentiation of reparative cells [23].

In the study conducted by Suh et al., significantly elevated levels of RANKL and an increased RANKL-to-osteoprotegerin (OPG) ratio were observed in patients with AIS. In both the AIS and control groups, a negative correlation was found between RANKL levels and the RANKL/OPG ratio with lumbar spine bone mineral density and serum OPG concentrations. Notably, no association was observed between RANKL, OPG, or the RANKL/OPG ratio and body mass index (BMI). These findings suggest that alterations in the RANKL/OPG axis may play a key role in the mechanisms underlying BMD loss in AIS patients, independent of body weight or BMI [24]. RANKL binds to its receptor RANK on the surface of osteoclast precursors, leading to their activation and increased bone resorption. Consequently, the weakening of spinal stabilizing structures may occur, potentially accelerating the progression of spinal curvature [25].

Additional support for inflammation-related bone involvement comes from studies on vitamin D–binding protein (DBP), an immunomodulatory molecule that serves as a precursor for Gc protein–derived macrophage activating factor (Gc-MAF). Gc-MAF enhances macrophage activation and osteoclast activity, linking immune responses with bone metabolism [26, 27]. Elevated DBP levels have been associated with greater curve severity in specific Lenke subtypes and were independently correlated with scoliosis progression. Although interactions between DBP, BMI, and AIS remain complex and incompletely understood, these findings suggest that immune–metabolic factors may influence bone remodelling and deformity progression [28, 29].

#### Immune dysregulation and paraspinal muscle remodelling

An increasing body of evidence suggests that inflammation may play a significant role in the development and progression of AIS—not only systemically, but also at the level of local paraspinal tissues. Histopathological and imaging studies indicate that the paraspinal muscles of AIS patients exhibit fibrotic changes and tissue remodeling, with these changes being more pronounced on the concave side of the spinal curvature. Fibrosis is a classical outcome of chronic immune system activation, particularly involving macrophages, which modulate tissue regeneration, degradation, and extracellular matrix deposition through the secretion of both pro- and anti-inflammatory mediators. However, earlier histochemical studies suggest that paraspinal muscle alterations in AIS may not reflect primary myopathic processes. In a classic biopsy-based study, paravertebral muscles in AIS patients demonstrated predominantly aerobic metabolic profiles without evidence of structural myopathy, supporting the concept that scoliosis-related muscle changes are secondary and locally driven rather than indicative of systemic muscle disease [30].

The study by Samaan et al. [10] conducted within the framework of the ICONS project (Immunometabolic Connections to Scoliosis) aimed to investigate in detail the immunological mechanisms underlying muscle fibrosis in AIS. The authors hypothesized that the concave side of the spinal curvature is characterized by increased infiltration of M2 macrophages with anti-inflammatory properties, which secrete factors such as TGF-β1. This cytokine stimulates fibro-adipogenic progenitors (FAPs) to proliferate and differentiate into fibroblasts, resulting in enhanced collagen deposition and reduced muscle elasticity. Concurrently, sustained activity of pro-inflammatory M1 macrophages—producing cytokines such as TNF-α, IL-1β, and IL-6—may promote a chronic inflammatory microenvironment, leading to repeated cycles of tissue damage and incomplete regeneration.

In the model proposed by the authors, AIS is conceptualized as a state of “acute-on-chronic” inflammation of the paraspinal muscles, triggered by repetitive microinjuries resulting from asymmetric spinal loading. This condition leads to the simultaneous presence of degradative and profibrotic signals. The outcome is a gradual loss of muscle function, disruption of biomechanical balance, and further progression of the spinal deformity. A key component of the ICONS study is the comparison of immune cell phenotypes, cytokine and chemokine expression, and FAP activation on both sides of the spinal curvature. The authors propose that identifying differences in the local inflammatory response may lead to the discovery of novel biomarkers of AIS progression and potential therapeutic targets, such as modulation of macrophage polarization or inhibition of excessive FAP activation.

Pilot data from the ICONS feasibility study demonstrated that while total macrophage numbers did not differ significantly between the concave and convex sides, macrophage abundance correlated positively with total body fat mass [31]. This finding suggests that systemic metabolic status may modulate local immune responses in paraspinal muscles, further influencing tissue remodelling and biomechanical imbalance in AIS.

#### Integration of inflammatory pathways across tissues

From a cellular perspective, other immune cell populations may also contribute to the pathogenesis of AIS. T lymphocytes, particularly the Th17 subpopulation, remain a focus of interest due to the role of IL-17 in modulating inflammatory responses and tissue remodelling, as previously described. An imbalance between proinflammatory Th17 cells and regulatory T cells (Tregs) could promote the persistence of chronic inflammation within the microenvironment of paraspinal tissues, although direct evidence in AIS remains limited and warrants further investigation. The role of B lymphocytes in the pathogenesis of AIS remains less well studied to date. In other conditions, such as rheumatoid arthritis or Paget’s disease, hyperactive B cells produce increased amounts of proinflammatory cytokines (e.g., TNF-α, IL-6) and RANKL, leading to chronic osteoclast activation and accelerated bone resorption. Concurrently, depletion of B cells (e.g., following rituximab treatment) may result in inhibition of bone loss, confirming their role in bone homeostasis. The authors also emphasize that B cells are an important component of the local immune response in the bone microenvironment – functioning as antigen-presenting cells that modulate T lymphocyte activity, potentially exacerbating chronic inflammation in periosteal tissues. By analogy, dysregulated B cell activity could potentially influence vertebral bone remodelling and paraspinal muscle inflammation in AIS; however, this hypothesis remains speculative and requires direct experimental validation [32, 33].

### The relationship between inflammation and structural changes in the spine

Chronic inflammation is a well-established driver of structural remodelling in musculoskeletal tissues, including vertebral bone, intervertebral discs, cartilage, and surrounding soft tissues. Although AIS is not classified as an inflammatory spinal disorder, insights from inflammatory and degenerative orthopedic conditions provide a valuable conceptual body for understanding how low-grade immune activation may influence spinal integrity. Importantly, in this chapter, findings derived directly from AIS studies are clearly distinguished from mechanisms inferred from other musculoskeletal or inflammatory conditions.

#### Inflammatory regulation of vertebral bone: evidence from AIS and related conditions

In inflammatory rheumatic diseases such as rheumatoid arthritis and ankylosing spondylitis, vertebral bone loss shows a strong correlation with inflammatory activity. Pro-inflammatory cytokines, particularly TNF-α and IL-6 promote osteoclastogenesis through upregulation of the RANKL/RANK signalling pathway while simultaneously inhibiting osteoblast-mediated bone formation [34–36]. These mechanisms illustrate the sensitivity of vertebral bone to immune-mediated regulation.

In AIS, direct evidence of inflammatory bone destruction is limited; however, several AIS-specific observations suggest a potential role for inflammation-related dysregulation of bone remodelling. Reduced bone mineral density, altered vertebral growth patterns and abnormalities in bone metabolism have been consistently reported in AIS populations. These findings raise the possibility that subclinical inflammatory signalling—operating below the threshold of overt systemic inflammation—may contribute to impaired vertebral strength and increased susceptibility to asymmetric mechanical loading during adolescent growth. At present, this relationship should be regarded as associative rather than causal.

#### Intervertebral disc and facet joint degeneration: distinguishing established and AIS-specific evidence

Activation of innate immune pathways, including Toll-like receptor (TLR) signalling, is a well-characterized mechanism driving intervertebral disc degeneration and cartilage breakdown in degenerative disc disease and osteoarthritis. In these conditions, TLR activation induces the production of pro-inflammatory cytokines such as IL-1β, IL-6, and TNF-α, leading to upregulation of matrix metalloproteinases and progressive extracellular matrix degradation [37–40]. These mechanisms are firmly established in non-AIS spinal pathologies and provide a biological model of inflammation-driven structural deterioration.

Direct evidence supporting similar processes in AIS remains limited; however, AIS-specific studies have begun to bridge this gap. Bisson et al. demonstrated degenerative changes in facet joint cartilage obtained from AIS patients that resemble early osteoarthritic alterations, including elevated expression of IL-1β and matrix-degrading enzymes [41]. In subsequent work, the same group reported a strong association between TLR expression and the production of pro-inflammatory cytokines and proteases within scoliotic facet joints [42]. These findings represent direct evidence from AIS tissues and suggest that innate immune activation and low-grade inflammation may contribute to premature degeneration of posterior spinal elements in AIS. Nevertheless, the extent to which these mechanisms parallel classical degenerative disc disease remains uncertain and requires further investigation.

#### Angiogenesis, neoinnervation, and neurogenic inflammation: hypothesis-generating evidence

Tissue degeneration, however, is not the only secondary effect of chronic inflammation. In inflamed tissues, angiogenesis and neoinnervation often occur in parallel. It has been shown that IL-1β and TNF-α induce the expression of vascular endothelial growth factor (VEGF), nerve growth factor (NGF), and brain-derived neurotrophic factor (BDNF), thereby promoting pathological neovascularization and nerve ingrowth within the affected intervertebral disc [43]. Both histopathological and MRI studies have confirmed the presence of neovascularization in patients with elevated cytokine expression in their intervertebral discs [44]. Although human-based studies remain limited, Qian-Yi Li et al. demonstrated increased VEGF expression on the convex side of experimentally induced scoliosis in a rat model [45]. These findings should be interpreted as hypothesis-generating, supporting the plausibility of inflammation-related vascular and neural remodelling in AIS rather than confirming their presence. Further histological and molecular studies in AIS tissues are needed to clarify the relevance of these mechanisms.

Damage to the intervertebral disc is also associated with the release of neuropeptides, such as substance P and calcitonin gene-related peptide (CGRP), from nociceptive nerve endings. These neuropeptides activate receptors on both disc cells and infiltrating leukocytes, leading to the further release of neurogenic mediators and nerve growth factors, thereby amplifying neoinnervation. This creates a positive feedback loop that exacerbates the inflammatory process [46].

Taken together, available evidence supports a model in which inflammation acts as a modulatory factor rather than a primary driver of structural changes in AIS. Data from inflammatory and degenerative spinal disorders demonstrate how immune activation can weaken vertebral bone, degrade disc and cartilage matrices, and promote angiogenesis and neoinnervation. AIS-specific studies, particularly those examining facet joint tissues, suggest that elements of these pathways may also be active in scoliosis, albeit at a lower intensity and in interaction with growth-related biomechanical forces. In AIS, inflammation is therefore best viewed as a process that may increase tissue vulnerability to asymmetric loading during adolescence, contributing to microstructural remodelling and curve progression.

### Gut microbiota, inflammation, and AIS – a new perspective

The gut microbiota (GM) plays a crucial role in regulating systemic physiological functions, extending well beyond the gastrointestinal tract. Growing evidence supports the existence of communication axes, such as the gut–skeletal axis, which links the gut to other body systems and modulates their function. Recent studies indicate that the commensal microbiota serves as a critical regulator of tissue remodelling and homeostasis in extra-intestinal tissues [47]. One of the first metabolites identified as a mediator of the gut–bone axis is insulin-like growth factor 1 (IGF-1), produced primarily in the liver in response to nutrient intake and regulated by GM. In a study by Jing Yan et al., IGF-1 was shown to promote bone growth and remodelling. In germ-free mice colonized with conventional microbiota but free of specific pathogens, serum IGF-1 levels were markedly elevated following microbial colonization, accompanied by a significant increase in IGF-1 production in the liver and adipose tissue. Conversely, treatment of conventional mice with antibiotics reduced serum IGF-1 levels and suppressed bone formation. Supplementation of antibiotic-treated mice with short-chain fatty acids (SCFAs)—microbial metabolites—restored both IGF-1 levels and bone mass to those observed in untreated controls [48, 49]. Another regulatory molecule is hydrogen sulfide (H₂S), whose concentration is strongly influenced by GM and which affects bone marrow-derived mesenchymal stem cells. In a study by Yi Liu et al., mice with homozygous cystathionine β-synthase (CBS) deficiency (CBS-/-) and approximately 66% of heterozygous CBS+/- mice with reduced H₂S levels exhibited osteopenic phenotypes, as confirmed by histopathological analysis showing reduced femoral bone volume, particularly in trabecular bone. Micro-CT analysis further revealed decreased BMD and lower bone tissue volume in the femurs [50].

The mechanisms underlying the gut–skeletal axis remain incompletely understood. However, in recent years, an increasing body of evidence suggests that the pathogenesis of AIS may be linked to GM imbalance, referred to as dysbiosis. Comparative and functional analyses have revealed consistent differences in the bacterial composition and metabolic potential of the gut microbiota in AIS patients compared to controls. These differences may influence the gut–bone axis and modulate chronic inflammation, contributing to reduced BMD and the progression of spinal deformities.

One of the first large-scale comparative studies [51] utilizing 16 S rRNA sequencing demonstrated that the GM of AIS patients significantly differs from that of both comparison groups — individuals with congenital scoliosis and healthy controls. In AIS, a notably lower Firmicutes/Bacteroidetes ratio was observed, along with a decrease in *Ruminococcus*, a genus of bacteria responsible for short-chain fatty acid (SCFA) production. At the same time, there was an increased abundance of *Bacteroides fragilis* and an upward trend in *Hungatella*. LDA/LEfSe analyses revealed an enrichment of *Parasutterella*, *Hungatella*, and *B. fragilis*, along with a depletion of *Parabacteroides*,* Romboutsia*, and *Dorea*. These findings were further validated in sibling-pair analyses, minimizing the confounding effects of genetic and environmental factors. Additionally, among AIS patients with low BMI (< 18.5), species richness (Chao1) was significantly reduced (*p* = 0.0046), and BMI positively correlated with microbial diversity only within this subgroup. This suggests a potential link between undernutrition, dysbiosis, and metabolic deficiencies in the pathogenesis of AIS.

In a separate study [52] involving 126 AIS patients (Cobb angle > 40°), the GM was analyzed in relation to BMD. Patients with osteopenia (femoral neck Z-score < − 1) exhibited lower alpha diversity (Shannon index) and higher abundances of *Escherichia–Shigella* and *Faecalibacterium*, along with a reduction in *Prevotella*. Notably, the abundance of *Escherichia–Shigella* was negatively correlated with BMD and positively correlated with the bone resorption marker β-CTX, whereas *Prevotella* showed the opposite trend. Functional predictions of microbial activity (Tax4Fun) revealed alterations in starch, sucrose, pyruvate, and sulfur amino acid metabolism pathways, which may affect SCFA production and bone homeostasis.

Nan Shen et al. further demonstrated that AIS patients exhibit a distinct gut microbiota profile even at the bacterial phylum level. Their analysis showed an increased abundance of *Prevotella*,* Gelria*, and *Desulfovibrio* in stool samples from AIS patients, whereas the abundance of *Parasutterella*,* Tyzzerella*, and *Phascolarctobacterium* was decreased. Additionally, the abundance of *Prevotella* was found to be positively correlated with the Cobb angle [53]. Similarly, Wen et al. observed an increased abundance of *Prevotella* in the stool of patients with ankylosing spondylitis, with comparable findings reported in HLA-B27 transgenic rat models [54], suggesting that this genus may play a role in immune response modulation. It has been hypothesized that *Prevotella* may activate the NF-κB pathway, thereby initiating an inflammatory response targeting joint tissues [55].

**Table 1:** Summary of gut microbiota alterations and associated clinical features in adolescent idiopathic scoliosis

Author (year)	Study population	Methodology	Key gut microbiota alterations	Main clinical associations	Key conclusions
Fang et al. (2025) [51]	AIS vs. congenital scoliosis vs. healthy controls; China	16 S rRNA sequencing, LEfSe, sibling-pair analysis	↓ Firmicutes/Bacteroidetes ratio; ↓ *Ruminococcus*; ↑ *Bacteroides fragilis*, *Hungatella*, *Parasutterella*	Lower microbial diversity in AIS with low BMI; BMI correlated with diversity only in AIS	AIS is associated with a distinct gut microbiota profile independent of genetic and environmental confounders
Li et al. (2024) [52]	126 AIS patients (Cobb angle > 40°); China	16 S rRNA sequencing, Tax4Fun functional prediction	↓ *Prevotella*; ↑ *Escherichia–Shigella*, *Faecalibacterium*	*Escherichia–Shigella* negatively correlated with BMD and positively with β-CTX	Gut microbiota dysbiosis is associated with aberrant bone homeostasis in AIS
Shen et al. (2019) [53]	AIS patients vs. controls; China	16 S rRNA sequencing, plasma proteomics	↑ *Prevotella*, *Desulfovibrio*; ↓ *Parasutterella*, *Phascolarctobacterium*	*Prevotella* positively correlated with Cobb angle	AIS is associated with a specific microbiota–proteome signature linked to curve severity
Lai et al. (2023) [56]	Genetic datasets (GWAS); AIS	Mendelian randomization	Protective taxa: *Bilophila*, *Eubacterium eligens*, *Prevotella9*; Risk taxa: *Mollicutes RF9*, *Catenibacterium*	Genetic association with AIS risk	Suggests potential causal role of specific microbial taxa in AIS
Zhao et al. (2024) [57]	Genetic datasets; AIS	Mendelian randomization	*Actinomycetaceae*, *Actinomycetales* associated with lower AIS risk	No direct mediation by classical cytokines	GM may influence AIS via metabolites and intestinal barrier modulation

Mendelian randomization (MR) studies have provided evidence for a potential causal relationship between gut microbiota composition and AIS. Taxa such as *Bilophila*,* Eubacterium eligens*,* Prevotella9*, and *Ruminococcus*2 exhibited a protective effect (lower odds ratios for AIS), whereas *Mollicutes* RF9, *Catenibacterium*, and *Ruminiclostridium6* were associated with an increased risk [56]. In another MR study, *Actinomycetaceae* and *Actinomycetales* were linked to a lower risk of scoliosis. Mediation analyses revealed that well-known pro-inflammatory cytokines do not directly mediate the GM→AIS pathway, suggesting that bacterial metabolites and modulation of the intestinal barrier may play a more prominent role [57]. The summary of gut microbiota alterations in AIS is listed in Table 1.

**Fig. 1:**
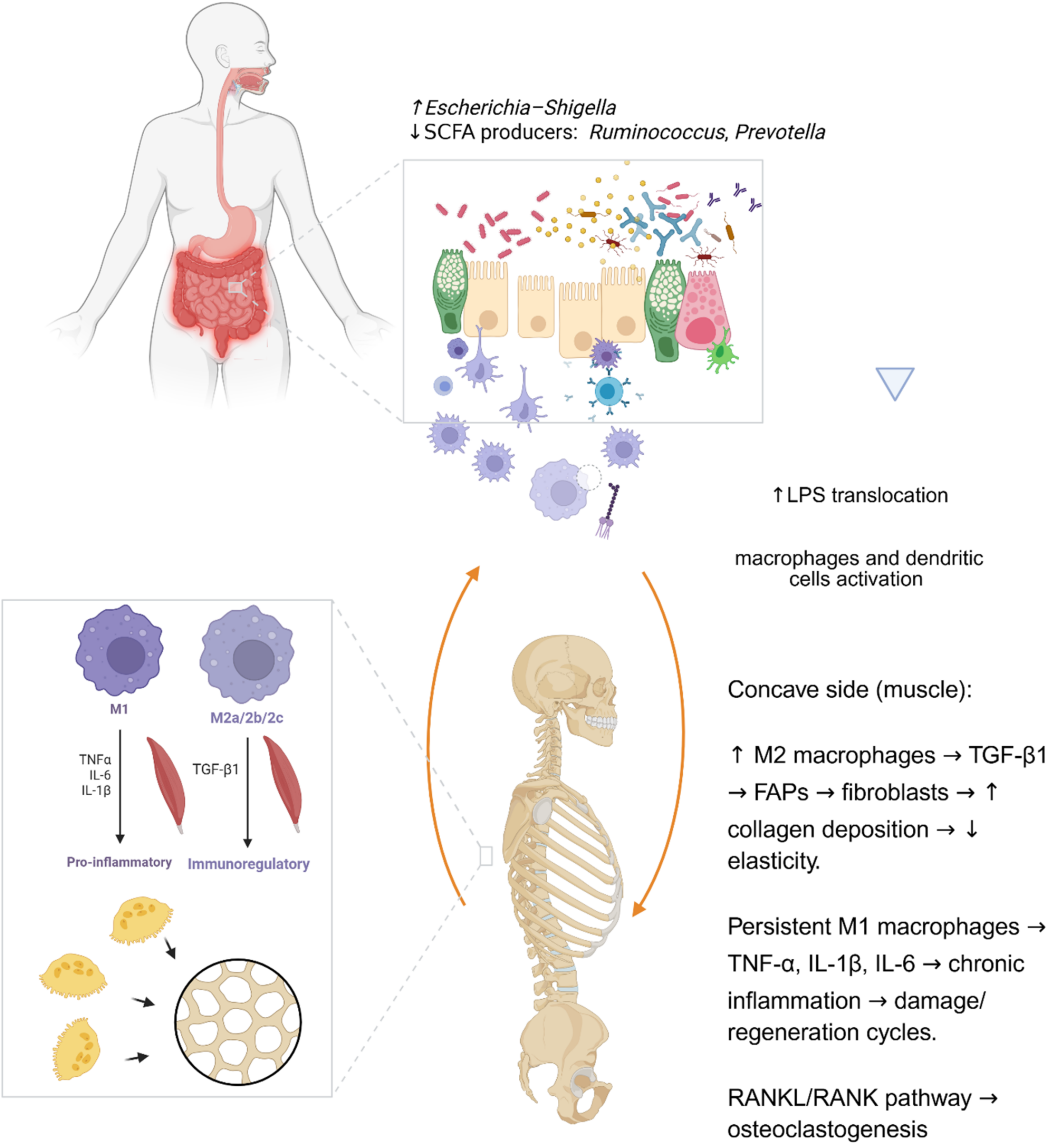
Proposed gut–muscle–bone axis in scoliosis pathogenesis. Dysbiosis characterized by increased *Escherichia–Shigella* and reduced SCFA-producing genera (*Ruminococcus*, *Prevotella*) promotes LPS translocation and immune cell activation. On the concave side of the spine, M2 macrophage–driven TGF-β1 signalling stimulates fibroblasts and collagen deposition, reducing elasticity, while persistent M1 macrophages maintain chronic inflammation through TNF-α, IL-1β, and IL-6, perpetuating tissue damage–repair cycles. Activation of the RANKL/RANK pathway contributes to osteoclastogenesis and bone remodelling

Considering the described studies and the hypotheses proposed by others [58] the mechanisms linking dysbiosis with inflammation and AIS may include the following (Fig. 1):

1. Reduced abundance of SCFA-producing bacteria (*Ruminococcus*,* Prevotella*) limits the availability of butyrate, propionate, and acetate — key metabolites with anti-inflammatory properties [59].
2. Decreased SCFA levels weaken the integrity of the intestinal barrier, facilitating the translocation of bacterial endotoxins: lipopolysaccharide (LPS) into the circulation.
3. LPS activates macrophages and dendritic cells, leading to increased production of IL-1β, IL-6, and TNF-α, which stimulate osteoclastogenesis via the RANKL/RANK pathway.
4. An increase in *Escherichia–Shigella* — a typical member of the *Proteobacteria* phylum — may further exacerbate endotoxemia and systemic inflammation.
5. *Bacteroides fragilis* and *Hungatella* may modulate bile acid metabolism and immune signaling, which, under conditions of dysbiosis, can promote chronic low-grade inflammation [60].

Given that the gut microbiota profile in AIS appears to differ not only from that of healthy individuals but also from patients with other spinal deformities, this suggests a distinct microbial signature specific to AIS. Dysbiosis, through modulation of the gut–bone axis, may influence BMD, bone tissue remodelling, and curve progression, representing a potential target for probiotic or prebiotic interventions in the future. Among bacteria with potential protective effects, genera such as *Bifidobacterium* and *Lactobacillus* have been highlighted for their anti-inflammatory properties, their role in enhancing vitamin D absorption, and their ability to suppress osteoclast activity, which may help mitigate bone mass loss [57, 61].

### Anti-inflammatory approaches in AIS: current evidence and future research directions

Current management of scoliosis, particularly advanced stages, remains largely based on mechanical and surgical approaches rather than targeted biological interventions. Surgical strategies primarily rely on spinal instrumentation systems aimed at deformity correction, stabilization, and control of curve progression. For example, magnetically controlled growing rods (MCGR) have been introduced to allow spinal growth while reducing the need for repeated surgical procedures. However, despite these technological advances, high rates of complications and implant-related failures have been reported, underscoring the limitations of current treatment strategies and the need for more mechanism-based therapeutic approaches [62, 63].

Increasing recognition of immune dysregulation and low-grade inflammation in AIS has prompted discussion about whether inflammatory pathways could represent future therapeutic targets. However, it is critical to emphasize that anti-inflammatory therapies are not currently part of standard AIS management, and no disease-modifying anti-inflammatory intervention has been validated in AIS. Most therapeutic concepts discussed in the literature are extrapolated from other spinal, musculoskeletal, or inflammatory conditions and should therefore be regarded as exploratory and hypothesis-generating rather than clinically actionable for AIS.

To date, AIS-specific interventional studies targeting inflammatory pathways are scarce. Unlike inflammatory spinal diseases, AIS does not present with overt systemic inflammation, and the inflammatory alterations described in AIS are subtle, heterogeneous, and incompletely characterized. Consequently, systemic anti-inflammatory pharmacotherapy—such as non-steroidal anti-inflammatory drugs (NSAIDs) or biological cytokine inhibitors targeting TNF-α or IL-17—cannot currently be justified for AIS outside of experimental research settings. Their established efficacy in conditions such as rheumatoid arthritis or ankylosing spondylitis demonstrates the therapeutic potential of inflammatory pathway modulation but does not imply direct translational relevance to AIS [64–66].

Biological therapies such as platelet-rich plasma (PRP) have demonstrated immunomodulatory and regenerative effects in degenerative disc disease models. Evidence suggests that leukocyte-poor PRP formulations may better support tissue regeneration while limiting inflammatory activation [26]. However, PRP has not been systematically investigated in AIS, and its relevance to scoliosis-related tissue remodelling remains speculative [67].

Probiotic supplementation and anti-inflammatory diets are gaining attention in research on chronic inflammatory conditions, including spinal diseases. The GM is a central focus of these interventions, as its dysregulation can significantly affect the immune system, cytokine balance, and chronic low-grade inflammation—factors that may also play a role in the pathogenesis of AIS [68–70]. Probiotics have been shown to modulate immune responses by reducing the production of pro-inflammatory cytokines (e.g., TNF-α, IL-6) and promoting the synthesis of anti-inflammatory cytokines (e.g., IL-10). Similarly, an anti-inflammatory diet, rich in fiber, polyphenols, and omega-3 fatty acids, contributes to an improved composition of the gut microbiota and the reduction of inflammatory markers [71, 72]. Although direct studies evaluating the efficacy of probiotic therapy and anti-inflammatory diets in the treatment of AIS are currently limited, a growing body of evidence highlights a link between gut microbiota, inflammation, and the regulation of the gut–brain–bone axis [73].

## Conclusions

Adolescent idiopathic scoliosis (AIS) remains a disorder of unclear and complex aetiology. Current evidence supports a multifactorial biological model in which chronic low-grade inflammation acts as a central integrator of systemic and local pathogenic processes. Altered cytokine signalling and immune cell imbalance may promote dysregulated bone remodelling via the RANKL/RANK pathway, while persistent inflammatory activation within paraspinal muscles contributes to fibrosis, impaired regeneration, and biomechanical asymmetry. In parallel, gut microbiota dysbiosis may further amplify inflammatory signalling through intestinal barrier dysfunction, reduced production of anti-inflammatory microbial metabolites, and activation of the gut–bone–muscle axis.

Although causal relationships remain to be fully established, these interconnected mechanisms provide a coherent framework linking immune dysregulation, musculoskeletal remodelling, and curve progression in AIS. Importantly, findings highlight opportunities for the identification of metabolic, inflammatory, and microbial biomarkers and support the development of integrative research strategies combining genetic risk stratification with multi-omic profiling. Future studies should prioritize large, longitudinal, and mechanistic approaches to validate these associations and translate them into predictive tools and targeted preventive or adjunctive interventions.

## Data Availability

No datasets were generated or analysed during the current study.
